# Efficacy of antithrombin in preclinical and clinical applications for sepsis-associated disseminated intravascular coagulation

**DOI:** 10.1186/s40560-014-0051-6

**Published:** 2014-12-31

**Authors:** Toshiaki Iba, Daizoh Saitoh

**Affiliations:** Department of Emergency and Disaster Medicine, Juntendo University, 2-1-1 Hongo, Bunkyo-ku, Tokyo 113-8421 Japan; Division of Traumatology, Research Institute, National Defense Medical College, Tokorozawa, Japan

**Keywords:** Antithrombin, Sepsis, Disseminated intravascular coagulation, Thrombin, Neutrophil extracellular traps, Damage-associated molecular pattern, Necrosis, Syndecan-4, Heparin, Protease-activated receptor

## Abstract

Antithrombin (AT) is known as an important physiological anticoagulant. AT inactivates thrombin and multiple other coagulation factors, thereby strongly inhibiting the over-activation of the coagulation system during disseminated vascular coagulation (DIC). AT also suppresses the pro-inflammatory reactions that are promoted through protease-activated receptor-1 during sepsis. One of the unique characteristics of AT is the conformational change it undergoes when binding to heparin-like molecules. The anticoagulant function is greatly accelerated after AT binds to externally administered heparin in the circulating blood. Meanwhile, AT also binds to syndecan-4 on the cell surface under physiological conditions, thereby contributing to local antithrombogenicity. The binding of AT and syndecan-4 upregulates prostaglandin I_2_ production, downregulates pro-inflammatory cytokine production, and suppresses the leukocyte-endothelial interaction. Other than these activities, recent preclinical studies have reported that AT might inhibit neutrophil necrotic cell death and the ejection of neutrophil extracellular traps. Together, these effects may lead to the attenuation of inflammation by decreasing the level of damage-associated molecular patterns. Although a number of animal studies have demonstrated a survival benefit of AT, the clinical benefit has long been argued since the effect of high-dose AT was denied in 2001 in a large-scale randomized controlled trial targeting patients with severe sepsis. However, recent clinical studies examining the effects of a supplemental dose of AT in patients with sepsis-associated DIC have revealed that AT is potentially effective for DIC resolution and survival improvement without increasing the risk of bleeding. Since DIC is still a major threat during sepsis, the optimal method of identifying this promising drug needs to be identified.

## Introduction

As was written in a recent review by Hunt [1], the fundamental strategy for caring for patients with sepsis-associated disseminated intravascular coagulation (DIC) is the management of underlying infection. During septic DIC, thrombus formation is driven by activated coagulation, the impairment of anticoagulant mechanisms including the antithrombin (AT) system, and compromised fibrin removal arising from the depression of the fibrinolytic system [2]. Microvascular thrombosis contributes to diminished oxygen delivery and subsequent organ dysfunction. Accordingly, anticoagulant therapy is expected to play some role in alleviating this dangerous condition [3]. In the early 2000s, some large-scale randomized controlled trials (RCTs) targeting severe sepsis were conducted [4-6], but none of the anticoagulants that were examined are currently available for clinical use. Actually, sepsis-associated DIC, instead of severe sepsis, might be an appropriate target for these anticoagulant therapies. Indeed, some subgroup analyses of subjects with sepsis-associated DIC in the above-mentioned RCTs have revealed effects on mortality [7,8]. However, the effects of these anticoagulants on septic DIC have not been examined in the well-qualified studies. Recently, a small-sized but properly designed RCT succeeded in demonstrating the efficacy of a physiological dose of AT for DIC resolution [9]. Following this report, an analysis using a nationwide administrative database in Japan revealed a positive effect of physiological AT use on mortality. Under these circumstances, the “Harmonized guidance for DIC” has been released by the International Society on Thrombosis and Haemostasis (ISTH) [10]. In this guidance, AT is graded as “potentially recommended”. Therefore, our present and future tasks will be to search for appropriate measures for AT use and to accumulate sufficient evidence.

## Review

### Preclinical evaluation

AT is a vitamin K-independent glycoprotein with a molecular weight of approximately 59 kDa and is one of the major natural anticoagulants that have been aggressively studied [8,11,12]. AT inhibits thrombin in a 1:1 fashion and leads to the formation of a thrombin-antithrombin complex (TAT), thereby inactivating the enzymatic activity of thrombin and leading to its elimination from the circulation. Therefore, the inactivation of thrombin is considered to be a rational therapeutic strategy for DIC. Apart from anticoagulation, the anti-inflammatory function of AT can also be explained by the neutralization of thrombin. Thrombin has been implicated in the inflammatory cascade [13]; specifically, it increases leukocyte rolling and adhesion [14] by increasing the expression of endothelial P- and E-selectin [15,16] and intercellular adhesion molecule-1 (ICAM-1) [16], thereby promoting leukocyte recruitment. Thrombin also elicits an inflammatory reaction through its receptor, protease activated receptor (PAR)-1, on the cell surface [17]. PAR-1 is known to play pivotal roles in the activation of inflammation [18] by inducing the production of pro-inflammatory cytokines and chemokines by the endothelium [16,19]. Thus, the anti-inflammatory function of AT is thought to depend, at least in part, on the blockage of the effects of PAR-1.

Another cell-modulating activity of AT is induced after binding with its specific receptor syndecan-4, a type of heparin-like glycosaminoglycan (GAG), on various types of cell surfaces [20]. One major property of AT is thought to be its ability to stimulate prostacyclin production by endothelial cells through the binding of AT and syndecan-4 [21]. Prostacyclin exerts an anti-inflammatory function by blocking neutrophil tethering on the vascular endothelium [22] and by downregulating the production of pro-inflammatory cytokines [23]. When AT and syndecan-4 bind together on neutrophils, monocytes, and lymphocytes, their interactions with the endothelium are suppressed. The downregulation of P-selectin may also be involved in the vessel wall integrity-preserving properties of AT [24].

**Figure 1:**
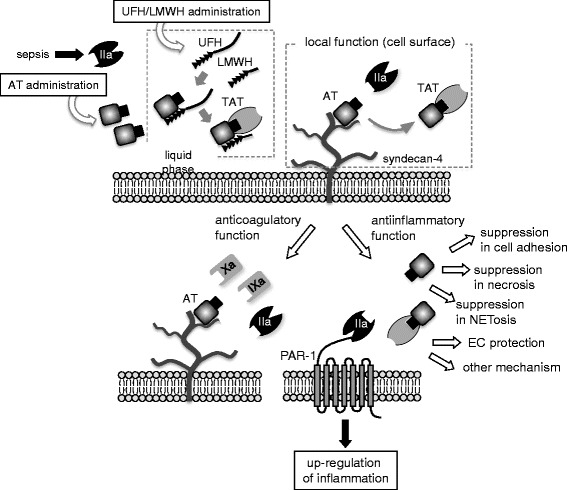
**Multifactorial functions of antithrombin in circulating blood and on the cell surface.** The interactions between antithrombin (*AT*) and the endothelium are shown in the figure. The affinity of antithrombin to thrombin and its enzymatic inhibition are increased by the binding of the heparin-binding site of AT to syndecan-4 on the cell surface or externally administered heparins. Thrombin loses its coagulant activity after the formation of a thrombin-antithrombin complex. Other than thrombin, AT inactivates factors Xa and IXa. As for its anti-inflammatory function, AT inactivates thrombin, thereby attenuating the cellular reactions through the activation of protease-activated receptor (*PAR*)-1.

The balance between the anticoagulation effect and the anti-inflammatory cellular function is dominantly regulated by heparins. AT contains a heparin-binding domain, and its anticoagulation activity is maximized by several orders of magnitude after binding with heparin in the bloodstream (Figure 1). This binding of heparin to the lysyl side chains of AT induces a nonreversible conformational change leading to a high affinity towards thrombin. Since the latter effect is more desired in sepsis, the coadministration of AT and heparin might not be a good choice for the treatment of patients with sepsis and not associated with DIC [7].

Recently, the mechanisms involved in the pathological derangement of coagulation in patients with sepsis have become increasingly clear. Tissue factor (TF) is a key factor in thrombin generation during sepsis [25]. However, recent studies have elucidated that damage-associated molecular patterns (DAMPs) from necrotic cells and neutrophil extracellular traps (NETs) and their components also play major roles in the activation of the coagulation system [26,27]. NETs activate coagulation by expressing tissue factor [28] and stimulate platelets via histones [29], which are also strong promoters of the extrinsic pathway [30]. Interestingly, recent studies have reported that antithrombin suppresses necrotic cell death and NET formation in the animal and *in vitro* studies [31,32].

In animal models, the effect of AT substitution on survival was first reported by Triantaphyllopoulos [33] in a lipopolysaccharide (LPS)-induced rabbit model of sepsis. Similar effects were reported in an *Escherichia coli*-induced primate model of sepsis by Taylor et al. [34]. Other than these reports, the protective effect of AT on mortality has been reported in various models of sepsis [35,36]. In our rat model, all the rats died when treated with a continuous infusion of 10.0 mg/kg of LPS over 24 h, but 50% of the rats survived when treated with low-dose AT and all the rats survived when treated with high-dose AT [37]. These results were adopted as the fundamental basis of the clinical studies.

### Clinical evaluation

A pharmacological dose of recombinant activated protein C [4], high-dose AT [5], and tissue factor pathway inhibitor [6] are widely known to have failed to demonstrate a survival benefit among patients with severe sepsis in large-scale RCTs. Some reasons have been suggested to explain the failures of these trials. First, even if the anti-inflammatory effects of anticoagulants have been advocated in animal and *ex vivo* studies, anticoagulant therapies seem to be effective only in the septic patients with DIC, but not in those without DIC, in a clinical setting. Subgroup analyses performed in subjects with DIC in the KyberSept trial [5] and PROWESS trial [38] revealed improvements in survival [7,8]. The second reason is that “pharmacological” or “high-dose” anticoagulants may cause bleeding. For example, treatment with high-dose AT was correlated with a significant hemorrhagic tendency. The incidence of total bleeding events was reported to be 12.8% in the control group and 22.0% in the AT-treated group (relative risk [RR], 1.71 [95% confidence interval [CI], 1.42–2.06]). Thus, increased bleeding events might diminish the beneficial effects of AT. Third, the concomitant use of heparin might interfere with the effect of AT. As a matter of fact, nearly 70% of high-dose antithrombin-treated patients received heparin. Hoffmann et al. [39,40] analyzed the data from KyberSept and reported increased bleeding risks associated with antithrombin plus concomitant heparin, compared with antithrombin alone. Regarding this issue, we will introduce the latest studies examining the above problems in the following section.

While a high dose can be harmful, an insufficient dose will be ineffective. So far, we have conducted two nonrandomized multi-institutional post-marketing surveys to determine the optimal AT dose. In the first survey, a total of 729 sepsis-associated DIC patients with an AT activity of 70% or lower were analyzed. Among these patients, AT was substituted at a dose of either 1,500 IU/day (*n* = 650) or 3,000 IU/day (*n* = 79) for three consecutive days. The dose selection was made by the attending physicians based primarily on each patient's condition, and the baseline AT activity level was lower among the patients who received 3,000 IU/day. As a result, the survival of the patients who had received 1,500 IU/day was 65.2%, while that of the patients who received 3,000 IU/day was 74.7%, and a logistic regression analysis showed that the supplemented AT dose of 3,000 IU/day contributed to a better survival outcome (odds ratio [OR], 1.912; *P* = 0.026) [41]. Whether this supplemental dose of AT increased the bleeding risk remained unclear because a placebo control was not included in this post-marketing survey. Nevertheless, since the incidence was 6.52% (major bleeding, 1.71%), which was even lower than that of the control group (major bleeding, 5.7%) in the KyberSept trial [5], we believe that AT supplementation for septic DIC patients is an acceptable treatment.

Since the survival difference did not reach statistical significance and the effect of AT was more prominent in severer cases (lower baseline AT activity) in the first survey, we conducted a second survey in septic DIC patients with baseline AT activities of less than 40%. A total of 307 patients (259 patients received 1,500 IU/day and 48 patients received 3,000 IU/day) were investigated in this second survey. The results demonstrated a significantly higher rate of DIC resolution (66.7% vs. 45.2%, *P* = 0.007) and a better survival outcome (77.1% vs. 56.4%, *P* = 0.010) among the patients supplemented with 3,000 IU/day of AT. Bleeding events were observed in 6.96% (major bleeding, 3.04%) of the patients supplemented with 1,500 IU/day and 6.52% (major bleeding, 4.35%) of the patients supplemented with 3,000 IU/day; this difference was not significant [42].

From these results, we assumed that AT supplementation at a sufficient dose could be expected to demonstrate a favorable effect. If so, which dose is most likely to be sufficient? The recovered AT activity level may provide a clue as to the most appropriate dose. The mean AT activity level in the patients supplemented with 3,000 IU/day recovered to within the normal range (>80%), while that of patients supplemented with 1,500 IU/day never reached the normal range in either survey in the second survey. In the first survey, the baseline AT activity was approximately 50%, and it rose up to above 80% in the patients supplemented with 1,500 IU/day. So, roughly 1,500 IU/day may be adequate if the initial AT activity level is above 50%; however, if the initial AT activity level is less than 50%, 3,000 IU/day will likely be needed. However, the Japanese health-care system allows the use of 3,000 IU/day of AT only in limited, severe cases. Thus, combination therapy with AT and recombinant thrombomodulin is now attracting attention [43].

Almost simultaneously with the above-mentioned surveys, the Japanese Association for Acute Medicine (JAAM) performed a multicenter placebo-controlled RCT in 60 septic DIC patients with baseline antithrombin levels of 50%–80%. The treatment group (30 cases) received a supplemental dose of AT (30 IU/kg/day × 3 days), and the mean AT activity recovered to 107.6% ± 24.5% in the treatment group, while it remained around 60% in the placebo group. The DIC resolution rate was 53.3% (16/30) in the treatment group, which was more than double that in the control group (20.0%, 6/30), and a significant improvement in DIC resolution was observed [9].

Following these reports, Tagami et al. [44] performed an analysis using a nationwide administrative database in Japan. A total of 9,075 patients with severe pneumonia and DIC were categorized into an antithrombin group (2,663 cases) and a control group (6,412 cases). Propensity score matching created a matched cohort of 2,194 pairs of patients with and without antithrombin treatment. The results demonstrated that standard AT supplementation (1,500–3,000 IU/day × 3 days) was associated with a 9.9% (95% CI, 3.5%–16.3%) reduction in 28-day mortality. Multiple logistic regression analyses showed an association between AT use and the 28-day mortality (adjusted odds ratio, 0.85 [95% CI, 0.75–0.97]). Based on the above-mentioned evidence, a supplemental dose of AT is assumed to be effective in patients with sepsis-associated DIC. However, to confirm this hypothesis, an adequately powered RCT will be required.

One topic in anticoagulant therapy is the development of recombinant AT. Only plasma-derived AT is presently available in Japan, but the development of recombinant AT is currently underway. A phase 3 trial has just been completed, and the results will be published in the near future.

### Usefulness as a biomarker

We have introduced the therapeutic characteristics of AT. However, AT can also be used as a biomarker for DIC. A reduced plasma level of AT in patients with DIC is a well-known fact [45]. Furthermore, decreased AT activity is reportedly correlated with the severity and outcome of patients [46]. Therefore, AT activity has become a popular test in patients with coagulopathy in Japan. The mechanisms responsible for the decrease in AT during sepsis are considered to be as follows: the consumption of AT during activated coagulation [47], the decreased synthesis of AT in the liver [47,48], the degradation of AT by neutrophil elastase [49,50], and the leakage of AT to the extravascular space. The reduced AT activity level results in a decreased ability to undergo thrombin inactivation, leading to the further acceleration of the coagulation system.

We previously reported that the AT activity level is approximately 80% of normal in septic patients without organ dysfunction, decreasing to approximately 60% in patients with severe sepsis, and 40% in patients with full-blown DIC [51]. Others have reported that the diagnostic value of an area under the receiver operating characteristic curve (AUC) of the AT activity exceeded 0.8 for the prediction of patient outcome [52,53]. Thus, we think that AT is an excellent discriminator of the severity of sepsis. Recently, Choi et al. [54] reported a significant correlation between AT and the DIC score in patients with sepsis, suggesting that AT is a good indicator of DIC severity. Indeed, AT had a significant prognostic power in a Kaplan-Meier analysis, showing a higher hazard ratio than conventional coagulation markers, such as D-dimer. Similar observations were reported in patients with conditions other than sepsis, such as multiple trauma or major surgery [45,55].

We conducted a multi-institutional observational study and analyzed samples from 78 sepsis patients with coagulopathy. Data collection was begun within 48 h after the platelet count first decreased to less than 150,000/mm^3^, and sequential changes in the coagulation markers, including AT activity, protein C activity, fibrin and fibrinogen degradation products (FDP), D-dimer, thrombin-antithrombin complex, plasmin α2-antiplasmin complex, soluble fibrin, and total plasminogen activator inhibitor-1, were evaluated. The results showed that the changes in hemostatic molecular markers were associated with the onset of organ dysfunction beginning at an early stage of sepsis and that AT activity and protein C activity exhibited the highest predictive values among these parameters [56]. Yanagida et al. [57] and Oshiro et al. [58] reported similar results for trauma patients.

Although the measurement of AT activity is valuable for estimating the severity and outcome of patients, it has not been included in major diagnostic criteria since the measurement of AT activity cannot be performed all day in many local laboratories [59]. In fact, all the major scoring systems consist of some of four routine laboratory tests: platelet count, prothrombin time (PT), fibrin-related marker level, and fibrinogen [60,61]. Although still controversial [62], Egi et al. [63] suggested that the inclusion of AT activity in the diagnostic criteria may provide a better diagnostic performance.

The other unique feature of antithrombin activity is its usefulness as a prognostic indicator after antithrombin supplementation. We evaluated the efficacy of measuring AT activity in 192 septic DIC patients supplemented with AT. A logistic regression analysis indicated that not only the baseline AT activity level but also the ΔAT activity level (the AT value on day 3 − the AT value on day 0) was related to patient outcome. Furthermore, the results revealed that an increase in AT activity had the largest contribution to patient survival (Table 1).

**Table 1:** **Relationship between outcome (28-day survival) and various factors using the stepwise method of logistic regression analysis**

	**Odds ratio**	***P*** **value**	**95% confidence interval**
Age	0.973	0.060	0.945–1.001
Body weight	1.032	0.071	0.997–1.068
Basement AT activity	1.042	0.006	1.012–1.073
ΔAT activity	1.031	0.001	1.012–1.050
Baseline fibrinogen	1.002	0.056	1.000–1.005
ΔSIRS score	0.681	0.008	0.512–0.906
Respiratory infection	0.490	0.061	0.232–1.035

*Δ* value on day 3 − value on day 0, *AT* antithrombin, *SIRS* systemic inflammatory response syndrome (original data).

In summary, the sensitivity and/or specificity of AT activity for predicting the morbidity and mortality of septic DIC is superior to that of global coagulation tests, and we recommend that AT activity be included in future diagnostic criteria for septic DIC. We also recommend examining sequential changes in this marker when supplementation therapy is performed. The baseline AT value may also help to determine appropriate candidates for anticoagulant therapy [41].

## Conclusions

AT, an important natural anticoagulant, inhibits over-activated coagulation and inflammation during sepsis through multifactorial pathways. However, its activity decreases significantly in sepsis-associated DIC. Although high-dose antithrombin administration has failed to provide a survival benefit, a supplementation dose that restores AT activity to within a normal range is expected to be useful. Currently, the “global guidance for the diagnosis and treatment of DIC” rates AT substitution as “potentially recommended,” meaning that AT can be used but that “further clinical study is required to prove its efficacy”.

## Abbreviations

APC: activated protein C
AT: antithrombin
AUC: area under the receiver operating characteristic curve
DAMPs: damage-associated molecular patterns
DIC: disseminated vascular coagulation
FDP: fibrin/fibrinogen degradation products
GAGs: glycosaminoglycans
ICAM-1: intercellular adhesion molecule-1
ISTH: International Society on Thrombosis and Haemostasis
JAAM: Japanese Association for Acute Medicine
LPS: lipopolysaccharide
NETs: neutrophil extracellular traps
PAR: protease-activated receptor
PT: prothrombin time
RCTs: randomized controlled trials
RR: relative risk
TAT: thrombin-antithrombin complex
TF: tissue factor
